# Prevalence of sarcopenic obesity in adult patients with diabetes and associated factors: a systematic review and meta-analysis

**DOI:** 10.3389/fnut.2026.1880656

**Published:** 2026-07-23

**Authors:** Zixue Gu, Changwei Mou, Feng Chen, Ke Zhou, Qingyun Li, Shan Fu, Zheng Shi, Shiyun Li, Rong Peng

**Affiliations:** 1 School of Basic Medical Sciences & School of Nursing, Chengdu University, Chengdu, Sichuan, China; 2 Clinical Medical College & Affiliated Hospital of Chengdu University, Chengdu University, Chengdu, Sichuan, China

**Keywords:** body composition, diabetes mellitus, diagnostic criteria, handgrip strength, muscle mass, obesity phenotype

## Abstract

**Objective:**

This systematic review and meta-analysis aimed to estimate the prevalence of sarcopenic obesity (SO) among adults with diabetes and summarize associated factors.

**Methods:**

Following the Preferred Reporting Items for Systematic Reviews and Meta-Analyses (PRISMA) guidelines, seven databases, including MEDLINE via PubMed, Cochrane Library, Embase, SinoMed, China National Knowledge Infrastructure (CNKI), Wanfang Database, and VIP, were searched from inception to 25 June 2026, for peer-reviewed observational studies published in English or Chinese. Two reviewers independently performed study selection, data extraction, and quality appraisal. For overlapping populations, the most recent and comprehensive report was retained. The pooled prevalence of SO was estimated using a random-effects model in Stata 18.0. Subgroup, sensitivity, and publication bias analyses were performed, and associated factors were summarized descriptively.

**Results:**

Thirty-four observational studies published between 2013 and 2026 were included. The pooled prevalence of SO among adults with diabetes was 24% (95% confidence interval: 19–29%), with substantial heterogeneity. Higher prevalence estimates were observed among community-dwelling patients (29%), female patients (28%), individuals in North America (29%), studies using bioelectrical impedance analysis for muscle mass measurement (25%), studies applying criteria based on muscle mass, muscle strength, and physical function (27%), and studies defining obesity by body mass index (25%) or waist circumference (23%). Reported factors associated with SO or SO-related body composition phenotypes included body mass index, fat mass index, limb fat mass, and A Body Shape Index ≥ 0.083; low serum irisin levels (<9.49 ng/mL) and poorer glycemic control or higher glycated hemoglobin levels; and older age, smaller calf circumference, lower handgrip strength, low physical activity, insulin use, and diabetes-related complications.

**Conclusion:**

Approximately one quarter of adults with diabetes are affected by SO, indicating a substantial clinical and public health burden. The prevalence of SO varied by patient source, sex, geographic region, assessment method, and diagnostic criteria, highlighting the need for standardized assessment. The reported associated factors suggest that SO is closely associated with adiposity, glycemic metabolism, physical function, and diabetes-related clinical characteristics. These findings support targeted screening, early identification, and integrated management to improve SO detection and reduce potential adverse outcomes.

**Systematic Review Registration:**

https://www.crd.york.ac.uk/PROSPERO/view/CRD420251274126, identifier PROSPERO (CRD420251274126).

## 1 Introduction

Sarcopenic obesity (SO), first proposed by Heber et al. in 1996, is a distinct clinical syndrome characterized by the simultaneous decline in skeletal muscle mass and function accompanied by excessive fat accumulation, rather than the mere coexistence of sarcopenia and obesity (1). With the aging global population and the rising prevalence of obesity, SO has emerged as an important clinical health concern and has garnered increasing attention (2). Individuals with SO bear a “double burden” of muscle loss and excess adiposity, placing them at higher risk of frailty, falls, functional decline, metabolic dysregulation, and all-cause mortality (3–5), with an estimated 24% higher risk of death compared to non-SO individuals (6).

Diabetes mellitus is one of the most prevalent chronic non-communicable diseases worldwide, and its prevalence continues to rise. According to projections from the International Diabetes Federation (IDF), the global number of individuals with diabetes is expected to reach 783 million by 2045 (7). The interplay between SO and diabetes has been increasingly recognized (8). Diabetes provides a metabolic milieu for the development of SO through insulin resistance, chronic low-grade inflammation, hormonal dysregulation, and lipid metabolism abnormalities, thereby accelerating disease progression (9). Accumulating evidence suggests that SO is not only highly prevalent in older and metabolically compromised populations but also confers a greater risk of adverse cardiometabolic outcomes, including impaired glucose homeostasis and incident type 2 diabetes, beyond the risk associated with sarcopenia or obesity alone (10). This synergistic risk may be driven by the complex crosstalk between adipose tissue and skeletal muscle, wherein dysfunctional adipokine signaling, chronic low-grade inflammation, and insulin resistance disrupt anabolic signaling pathways, leading to impaired muscle protein synthesis and enhanced proteolysis (11, 12). Evidence indicates that the presence of SO may further increase the risk of type 2 diabetes. This association is likely mediated by reduced muscle mass impairing glucose utilization, while excess adipose tissue activates chronic inflammatory pathways. The resulting muscle loss diminishes glucose uptake, and dysfunctional adipose tissue-muscle crosstalk promotes chronic inflammation and insulin resistance through inflammation and impaired insulin signaling, establishing a self-perpetuating metabolic imbalance (13).

Although previous studies have reported the prevalence of SO among patients with diabetes, existing evidence indicates an overall prevalence of approximately 27% (14). However, the recent literature over the past 3 years has not been systematically synthesized, and associated factors have not been comprehensively analyzed, which limits the ability to identify high-risk populations and inform targeted interventions. Furthermore, previous studies have not fully examined variations in patient sources, diagnostic criteria for SO, or associated factors. Therefore, the present study aims to conduct a systematic review and meta-analysis to comprehensively evaluate the prevalence of SO and factors associated with SO among adults with diabetes across different clinical and community settings, providing evidence for the identification of high-risk populations, optimization of chronic disease management, and development of targeted intervention strategies.

## 2 Materials and methods

This systematic review and meta-analysis were conducted in accordance with the Preferred Reporting Items for Systematic Reviews and Meta-Analyses (PRISMA) 2020 guidelines (15). The protocol was registered in the International Prospective Register of Systematic Reviews (PROSPERO; CRD420251274126). A completed PRISMA 2020 checklist is provided in Supplementary Table 1. The literature search was updated and re-run in all databases up to 25 June 2026, before the final analysis. For studies using overlapping populations or the same cohort, the most recent and comprehensive report was retained to avoid double-counting participants. When multiple prevalence estimates of SO were reported within the same study using different obesity definitions, only one estimate was included in the primary meta-analysis to avoid double-counting participants from the same study population.

### 2.1 Literature search

A comprehensive literature search was conducted in seven databases, including MEDLINE via PubMed, the Cochrane Library, Embase, SinoMed, CNKI, Wanfang Database, and VIP, from database inception to 25 June 2026. The review question and eligibility criteria were structured according to the population, index condition, outcome, and study design (PIOS) framework, which comprises population, index condition, outcome, and study design. To maximize search sensitivity, the electronic search strategy primarily combined terms related to the population and index condition, namely, diabetes- and SO-related terms, while the outcome and study design-related eligibility criteria were applied during title and abstract screening and full-text assessment according to the predefined eligibility criteria. Controlled vocabulary terms and free-text terms were adapted for each database. The final search was updated and re-run in all seven databases before the final analysis. In addition, the reference lists of previous systematic reviews and meta-analyses on related topics were manually screened to identify potentially eligible studies. The detailed database-specific search strategies are presented in Supplementary Tables 2–8.

### 2.2 Inclusion and exclusion criteria

The inclusion criteria were as follows: 1. peer-reviewed observational studies published in English or Chinese, including cross-sectional, case–control, or cohort studies; 2. participants were adults aged 18 years or older with type 1 or type 2 diabetes mellitus; 3. a clear definition of SO was provided; 4. sufficient data were available to calculate the prevalence of SO among adults with diabetes; and 5. studies were conducted in community, primary care, outpatient, or hospital settings. Studies in which the diabetes type was not specified were considered eligible only when the study population consisted of non-pregnant adults with diabetes and extractable prevalence data were available.

The exclusion criteria were as follows: 1. duplicate publications or studies using overlapping data, in which case the most complete report was retained; 2. studies involving gestational diabetes, non-diabetic populations, participants younger than 18 years, patients with end-stage organ failure, or patients with severe kidney disease; 3. studies involving mixed populations from which eligible data for adults with diabetes could not be extracted separately; 4. studies with incomplete or unavailable prevalence data; and 5. non-original articles, including systematic reviews, case reports, protocols, animal studies, and conference abstracts.

### 2.3 Study selection

All retrieved records were imported into EndNote X9, and duplicate records were removed. Two reviewers independently screened the titles and abstracts of all records according to the predefined eligibility criteria. Records that were clearly irrelevant were excluded at this stage. The full texts of potentially eligible studies were then independently assessed by the same two reviewers to determine final eligibility. Any disagreements were resolved through discussion or consultation with a third reviewer. Reasons for exclusion at the full-text screening stage were recorded and summarized in the PRISMA flow diagram.

### 2.4 Data extraction

Data were extracted independently by two reviewers using a standardized data extraction form. The extracted information included the first author, publication year, country or region, study design, study setting, type of diabetes, sample size, sex distribution, mean age, mean body mass index, the body composition assessment method, diagnostic criteria for SO, the number of participants with SO, the prevalence of SO, and reported factors associated with SO when available. Any discrepancies in data extraction were resolved through discussion or consultation with a third reviewer.

When multiple prevalence estimates of SO were reported within the same study using different obesity definitions, only one estimate was included in the primary meta-analysis to avoid double-counting the same study population. In such cases, the estimate based on body fat percentage (BFP) was preferentially selected when available, because it directly reflects adiposity and represents a body composition-based obesity indicator. Alternative estimates derived from the same study population were not included in the primary pooled analysis. Because the reported associated factors were limited and inconsistently reported across the included studies, they were summarized descriptively rather than quantitatively pooled.

### 2.5 Study quality appraisal

Two reviewers independently evaluated the methodological quality of the included observational studies using the Agency for Healthcare Research and Quality (AHRQ) checklist (16). The AHRQ checklist used for methodological quality appraisal is provided in Supplementary Table 9. Disagreements were resolved through discussion or consultation with a third reviewer. The AHRQ checklist consists of 11 items, and each item was scored as 0 or 1. The total score ranges from 0 to 11, with higher scores indicating better methodological quality. Studies with scores ≥8 were considered high quality, studies with scores of 6–7 were considered moderate quality, and studies with scores ≤5 were considered low quality.

### 2.6 Data analysis

Stata 18.0 software was used to pool prevalence estimates and calculate 95% confidence intervals (CIs). Heterogeneity was assessed using Cochran’s Q test and quantified using the *I^2^* statistic. A fixed-effect model was used when heterogeneity was low, defined as a *p-*value of >0.05 and *I^2^* of <50%; otherwise, a random-effects model was applied. Subgroup analyses were performed to explore the possible sources of heterogeneity. Sensitivity analyses were conducted using the leave-one-out approach to assess the stability of the pooled estimates and to examine whether the overall result was driven by any single study. Publication bias was evaluated using funnel plots and Egger’s regression test. A *p-*value of <0.05 was considered statistically significant.

### 2.7 Certainty of evidence assessment

The certainty of evidence for the pooled prevalence estimate was assessed using an adapted Grading of Recommendations, Assessment, Development and Evaluation (GRADE) approach for prevalence evidence. The assessment considered five domains: risk of bias, inconsistency, indirectness, imprecision, and publication bias. Risk of bias was judged according to the methodological quality of the included studies, assessed using the AHRQ checklist. Inconsistency was evaluated based on the magnitude of statistical heterogeneity and the consistency of prevalence estimates across studies and subgroups. Indirectness was assessed by considering the applicability of the study population, study settings, body composition assessment methods, and diagnostic criteria to the review question. Imprecision was evaluated according to the width of the 95% CI around the pooled prevalence estimate. Publication bias was assessed using funnel plots and Egger’s regression test. The overall certainty of evidence was rated as high, moderate, low, or very low.

## 3 Results

### 3.1 Study selection

**Figure 1:**
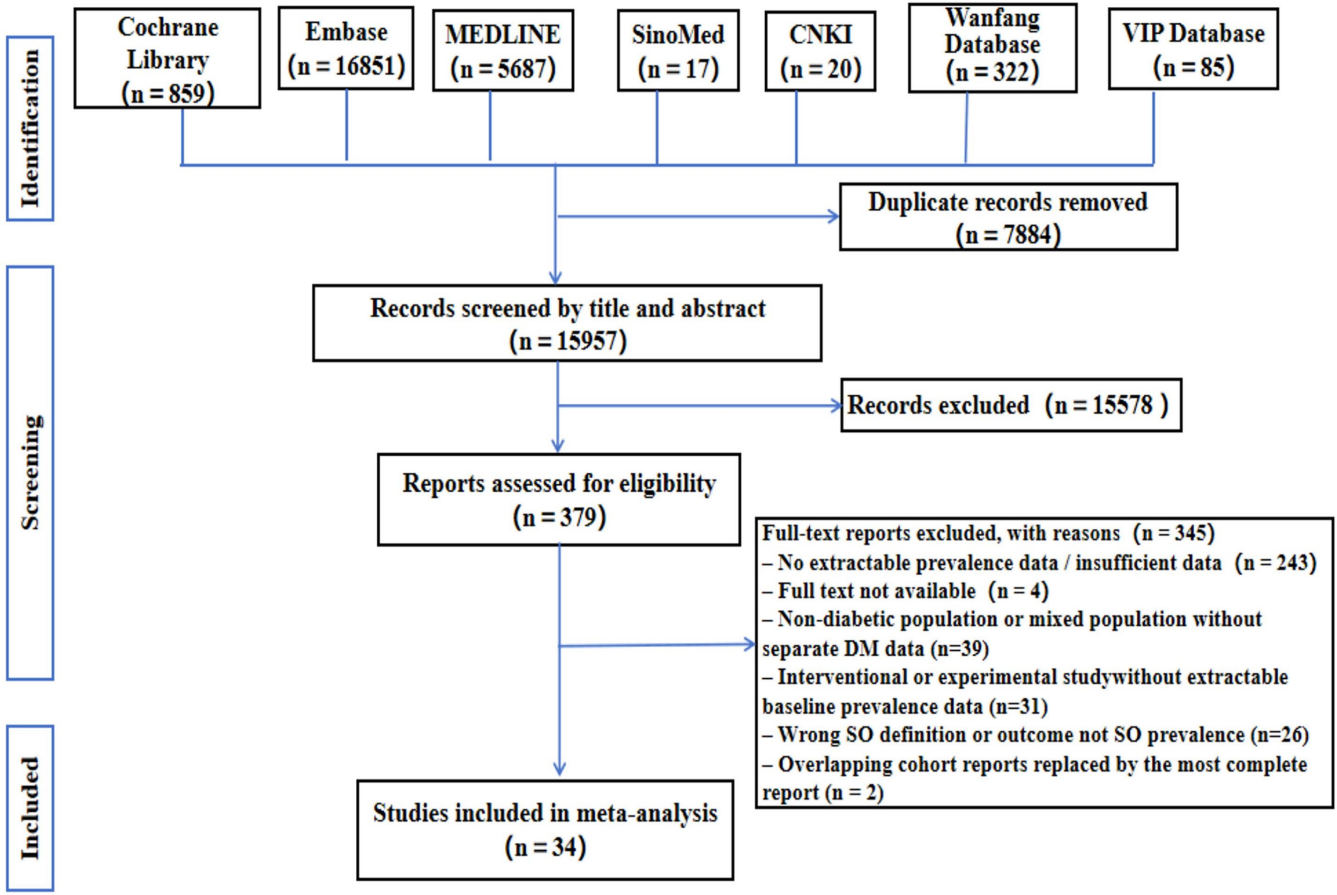
Flow diagram of the studies included in the systematic review. DM, diabetes mellitus; SO, sarcopenic obesity.

The study selection process is shown in Figure 1. A total of 23,841 records were identified from seven databases. After the removal of 7,884 duplicates, 15,957 records were screened based on their titles and abstracts, and 379 full-text articles were assessed for eligibility. In the updated search conducted up to 25 June 2026, six additional studies met the eligibility criteria. During reassessment of potentially overlapping populations, two reports derived from the KAMOGAWA-DM cohort were not retained in the primary prevalence meta-analysis, and the most recent and comprehensive report from this cohort was retained to avoid double-counting participants. Finally, 34 studies were included in the quantitative synthesis.

### 3.2 Study characteristics and methodological quality

The characteristics of the 34 included studies are summarized in Table 1.

**Table 1:** Characteristics of 34 included studies.

Author/year	Country	Design	Setting	Types of diabetes	Sample size (M/F)	Mean age	Mean BMI	Assessment method	Definition of SO		Prevalence SO (%)	Quality assessment
									Sarcopenia	Obesity		
Lin-2026 (17)	China	Cross-sectional	Community	T2DM	235 (99/136)	80.7 ± 4.3	25.2 ± 3.6	BIA	ASMI (M < 7.0 kg/m^2^, F < 5.7 kg/m^2^) HGS (M < 28 kg, F < 18 kg) or 6 MWT < 1.0 m/s or 5 STS ≥ 12 s	BFP (M ≥ 25%, *F* ≥ 30%)	35.00	6
Chen XY-2026 (18)	China	Cross-sectional	Inpatient	T2DM	580 (350/230)	52.00 ± 7.41	23.88 ± 3.73	DXA	ASMI (M < 7.23 kg/m^2^, F < 5.67 kg/m^2^)	Main extraction based on SO - BFP: BFP (M ≥ 25%, *F* ≥ 35%)	18.30	8
Limpaarayakul-2026 (19)	Thailand	Cross-sectional	Outpatient	T2DM	329 (148/181)	69.65 ± 7.39	26.09 ± 4.58	BIA	ASMI (M < 7.0 kg/m^2^, F < 5.7 kg/m^2^) HGS (M < 28 kg, F < 18 kg) or 6 MWT < 1.0 m/s or 5 STS ≥ 12 s	BFP (M ≥ 25%, F ≥ 35%)	16.40	6
Salman-2026 (20)	India	Cross-sectional	Outpatient	T2DM	151 (62/89)	57.2 ± 8.4	31.4 ± 3.9	DXA	ASMI (M < 7.0 kg/m^2^, F < 5.4 kg/m^2^) HGS (M < 28 kg, F < 18 kg) or 6 MWT < 1.0 m/s	BMI ≥ 25 kg/m^2^, WC (M ≥ 90 cm, *F* ≥ 80 cm), and BFP (M:≥ 25%, *F* ≥ 30%)	44.00	5
Yamamoto-2026 (21)	Japan	Cohort study	Outpatient	T2DM	799 (474/325)	68.6 ± 10.4	24.4 ± 4.4	BIA	HGS (M < 28 kg, F < 18 kg) and ALM/BMI (M < 0.789, F < 0.512)	BMI ≥ 25 kg/m^2^ and either VFA ≥ 100 cm^2^ or BFP (M ≥ 20%, F ≥ 30%)	3.00	8
Zou S-2025 (40)	China	Cross-sectional	Inpatient	Unspecified	198	18–70	NR	BIA	SMI (M < 7.0 kg/m^2^, F < 5.7 kg/m^2^), HGS (M < 28 kg, F < 18 kg)	BFP (M ≥ 25%, F ≥ 35%)	11.10	4
Bi-2024 (22)	China	Cross-sectional	Inpatient	T2DM	189 (113/76)	62.18 ± 11.71	25.96 ± 3.92	DXA	ASMI (M < 7.0 kg/m^2^, F < 5.4 kg/m^2^)	BFP (M ≥ 28%, *F* ≥ 40%)	33.97	7
Kim-2024 (23)	Korea	Cross-sectional	Community	Unspecified	1,586 (721/865)	75.4 ± 0.3	26.6 ± 0.2	HGS	HGS (M < 28 kg, F < 18 kg)	WC (M ≥ 90 cm, *F* ≥ 85 cm)	19.79	6
Xia W-2024 (41)	China	Cross-sectional	Community	Unspecified	287	≥45	NR	BIA	ASMI (M < 7.0 kg/m^2^, F < 5.7 kg/m^2^), HGS (M < 28 kg, F < 18 kg)	BFP (M ≥ 25%, F ≥ 30%)	11.10	5
Yogesh-2024 (24)	India	Cross-sectional	Outpatient	T2DM	250 (151/99)	65.2 ± 5.8	24.5 ± 3.2	BIA	ASMI (M < 7.0 kg/m^2^, F < 5.70 kg/m^2^), HGS (M < 28 kg, F < 18 kg), 6 MWT < 0.80 m/s	BMI ≥ 25 kg/m^2^	39.60	7
Yu BY-2024 (42)	China	Cross-sectional	Community	Unspecified	912	≥45	NR	HGS	HGS (M < 28 kg, F < 18 kg)	BMI ≥ 25 kg/m^2^	12.50	7
Zhou Y-2024 (43)	China	Cross-sectional	Inpatient	Unspecified	198	18–70	NR	BIA	SMI (M < 7.0 kg/m^2^, F < 5.7 kg/m^2^), HGS (M < 28 kg, F < 18 kg)	BFP (M ≥ 25%, F ≥ 35%)	11.10	4
Altinkaynak-2023 (25)	Turkey	Cross-sectional	Nursing home	Unspecified	63 (32/31)	76.7 ± 8.2	26.4 ± 5.5	HGS	HGS (M < 27 kg, F < 16 kg)	BMI ≥ 30 kg/m^2^	13.30	7
Sun L-2023 (26)	China	Cross-sectional	Inpatient	T2DM	543 (269/274)	67.66 ± 6.99	25.66 ± 3.62	DXA	ASMI (M < 7.0 kg/m^2^, F < 5.40 kg/m^2^), HGS (M < 28 kg, F < 18 kg), 6 MWT < 1.0 m/s	BFP (M ≥ 27%, F ≥ 40%)	5.70	6
Zhang Y-2023 (27)	China	Cross-sectional	Inpatient	Unspecified	50 (20/30)	≥65	NR	BIA	ASMI (M < 7.0 kg/m^2^, F < 5.7 kg/m^2^) HGS (M < 26 kg, F < 18 kg) or 6 MWT < 1.0 m/s or 5 STS ≥ 12 s or SPPB < 9	WC (M ≥ 90 cm, F ≥ 85 cm)	48	5
Zhang XX-2023 (28)	China	Cross-sectional	Inpatient	T2DM	1,112 (641/471)	53.45 ± 10.71	22.5 ± 1.5	BIA	ASM/Wt × 100% (M ≤ 32.2%, F ≤ 25.5%)	VFA ≥ 100 cm^2^	17.40%	6
Deng ZD-2022 (44)	China	Cross-sectional	Inpatient	Unspecified	294	≥60	NR	BIA	ASMI (M < 7.0 kg/m^2^, F < 5.7 kg/m^2^), HGS (M < 28 kg, F < 18 kg) or 6 MWT < 1.0 m/s	WC (M ≥ 90 cm, F ≥ 85 cm)	20.40%	6
Han-2022 (29)	China	Cross-sectional	Inpatient	T2DM	488 (268/220)	62–76	23.5 ± 2.0	BIA	ASMI (M < 7.0 kg/m^2^, F < 5.70 kg/m^2^)	BFP (M ≥ 25%, F ≥ 35%)	14.50%	7
Guo YS-2021 (45)	China	Cross-sectional	Outpatient	Unspecified	74	≥60	NR	BIA	ASMI (M < 7.0 kg/m^2^, F < 5.70 kg/m^2^), HGS (M < 26 kg, F < 18 kg), 6 MWT < 0.80 m/s	BFP (M ≥ 25%, F ≥ 35%)	16.20%	7
Oguz-2021 (30)	Turkey	Cross-sectional	Outpatient	T2DM	90 (20/70)	55.01 ± 8.81	31.58 ± 3.84	BIA	FM/FFM > 0.47 if BMI: 25–29.9 kg/m^2^; FM/FFM > 0.59 if BMI: 30–39.90 kg/m^2^		35.60%	6
Yin-2021 (46)	China	Cross-sectional	Community	Unspecified	744	56.75 ± 9.76	NR	BIA	ASMI (M < 7.0 kg/m^2^, F < 5.70 kg/m^2^)	BFP (M ≥ 25%, F ≥ 30%)	72.40%	6
Fukuda-2020 (31)	Japan	Cross-sectional	Outpatient	T2DM	745 (399/346)	64.6 ± 11.8	NR	DXA	ASMI (M < 7.0 kg/m^2^, F < 5.4 kg/m^2^)	A/G (M > 0.80, F > 0.62)	11.40%	7
Low-2020 (32)	Singapore	Cross-sectional	Outpatient	T2DM	1,235 (641/594)	61.6 ± 7.7	32.7 ± 5.0	BIA	FM/FFM > 0.80		19.35%	6
He QH-2019 (33)	China	Cross-sectional	Outpatient	T2DM	1,125 (586/539)	≥50	NR	BIA	ASMI (M < 7.18 kg/m^2^, F < 5.73 kg/m^2^), HGS (M < 29.5 kg, F < 21.2 kg)	BFP (M > 26.70%, F > 36%)	7.60%	8
Kim-2019 (34)	Korea	Cross-sectional	Inpatient	T2DM	233 (122/111)	57.8 ± 12.5	27.5 ± 1.4	DXA	ASMI (M < 7.0 kg/m^2^, F < 5.40 kg/m^2^)	WC (M ≥ 90 cm, F ≥ 85 cm)	8%	7
Wang MZ-2019 (47)	China	Cross-sectional	Inpatient	Unspecified	149	≥60	32.36 ± 1.89	CC	CC (M ≤ 30 cm, *F* ≤ 29 cm), 6 MWT < 0.8 m/s, HGS (M < 26 kg, F < 18 kg)	BMI ≥ 28 kg/m^2^	24%	7
Yasemin-2019 (35)	USA	Cross-sectional	Outpatient	Unspecified	602 (244/358)	60.2 ± 10.6	NR	BIA	Muscle mass (kg)/weight (kg) × 100 (M ≤ 37%, *F* ≤ 28%) and HGS (M < 30 kg, F < 20 kg)	BMI > 30 kg/m^2^	16.28%	4
Gomez-Peralta-2018 (36)	Spain	Cross-sectional	Outpatient	T2DM	199 (99/100)	M: 57 ± 10.70; F: 61.50 ± 12.2	36.4 ± 6.5	BIA	FM/FFM > 0.80		35.10%	7
Lim-2018 (37)	Korea	Cross-sectional	Community	Unspecified	340 (192/148)	69.70 ± 8	NR	DXA	ASM/Wt < 2 SD for healthy young adults	WC (M > 90 cm, F > 85 cm)	25.60%	6
Xiao-2018 (48)	Canada	Cross-sectional	Outpatient	Unspecified	70	54.60 ± 10.10	NR	BIA	FMI/FFMI > 95% of sex, BMI, and ethnicity-specific population-representative references		48.60%	5
Kang-2017 (38)	China	Cross-sectional	Community	Unspecified	550 (0/550)	>60	NR	DXA	ASM/Wt < 1 SD of the mean of the reference group	BMI ≥ 25 kg/m^2^	30%	7
Batsis-2016 (49)	USA	Cross-sectional	Community	Unspecified	1,060	≥60	NR	DXA	ALM: BMI ratio (M < 0.79, F < 0.51)	BFP (M ≥ 25%, F ≥ 35%)	29.90%	7
Ma-2016 (50)	USA	Cross-sectional	Community	Unspecified	159	71.80 ± 7.60	NR	24-h urinary creatinine	24 h-UC < median	BMI ≥ 30 kg/m^2^	25.20%	8
Lu-2013 (39)	China	Cross-sectional	Community	Unspecified	57 (16/41)	63.6 ± 10.1	NR	BIA	Skeletal muscle mass (kg)/weight (kg) × 100 (M ≤ 37%, *F* ≤ 27.80%)	BMI ≥ 25 kg/m^2^	30%	5

5 STS, five-time sit-to-stand test; 6 MWT, six-meter walking test; A/G, android-to-gynoid fat ratio; AHRQ, Agency for Healthcare Research and Quality; ALM, appendicular lean mass; ASM, appendicular skeletal muscle mass; ASMI, appendicular skeletal muscle mass index; BFP, body fat percentage; BIA, bioelectrical impedance analysis; BMI, body mass index; CC, calf circumference; DXA, dual-energy X-ray absorptiometry; F, female; FFM, fat-free mass; FFMI, fat-free mass index; FM, fat mass; FMI, fat mass index; HGS, handgrip strength; M, male; NR, not reported; SMI, skeletal muscle index; SO, sarcopenic obesity; SPPB, short physical performance battery; T2DM, type 2 diabetes mellitus; UC, urinary creatinine; VFA, visceral fat area; WC, waist circumference; Wt, weight.

The included studies were published between 2013 and 2026 and included a combined sample size of 15,696 participants. Sex-specific data were available in 23 studies (17–39), covering 11,551 of 15,696 participants, including 5,667 male (49.1%) and 5,884 female (50.9%) individuals. Eleven studies (40–50) did not report sex distribution. The included studies were conducted in ten countries or regions, including China (*n* = 18) (17, 18, 22, 26–29, 33, 38–47), the United States (*n* = 3) (35, 49, 50), Korea (*n* = 3) (23, 34, 37), Turkey (*n* = 2) (25, 30), Japan (*n* = 2) (21, 31), India (*n* = 2) (20, 24), Spain (*n* = 1) (36), Singapore (*n* = 1) (32), Canada (*n* = 1) (48), and Thailand (*n* = 1) (19). A majority of the studies were cross-sectional in design, and one study used a cohort design. The included studies varied in study setting, diabetes type, body composition assessment method, and diagnostic criteria for SO. For body composition or sarcopenia-related assessment, bioelectrical impedance analysis (BIA) was used in 20 studies (17, 19, 21, 24, 27–30, 32, 33, 35, 36, 39–41, 43–46, 48), and dual-energy X-ray absorptiometry (DXA) was used in 9 studies (18, 20, 22, 26, 31, 34, 37, 38, 49). The sarcopenia component of the SO definition varied across the included studies. Based on the extracted diagnostic criteria, 14 studies used a multidomain definition combining low muscle mass or body composition-derived muscle indices with muscle strength and/or physical performance, most commonly handgrip strength (17, 19, 20, 24, 26, 27, 33, 35, 40, 41, 43–45, 47). Physical performance measures were incorporated in fewer studies, including the six-meter walking test, five-time sit-to-stand test, or short physical performance battery. Three studies relied primarily on handgrip strength alone (23, 25, 42). Other studies defined the sarcopenia component using muscle mass-related, body composition-derived, or other surrogate indicators. As shown in Table 1, the obesity component of the SO definition was primarily based on body mass index (BMI), waist circumference (WC), body fat percentage (BFP), visceral fat area (VFA), or other body composition indicators across the included studies. The AHRQ quality assessment showed that four studies were rated as high quality (18, 21, 33, 50), 22 studies as moderate quality (17, 19, 22–26, 28–32, 34, 36–38, 42, 44–47, 49), and 8 studies as low quality (20, 27, 35, 39–41, 43, 48). The detailed item-level quality assessment results are presented in Supplementary Table 10.

### 3.3 Pooled prevalence and subgroup analyses

**Figure 2:**
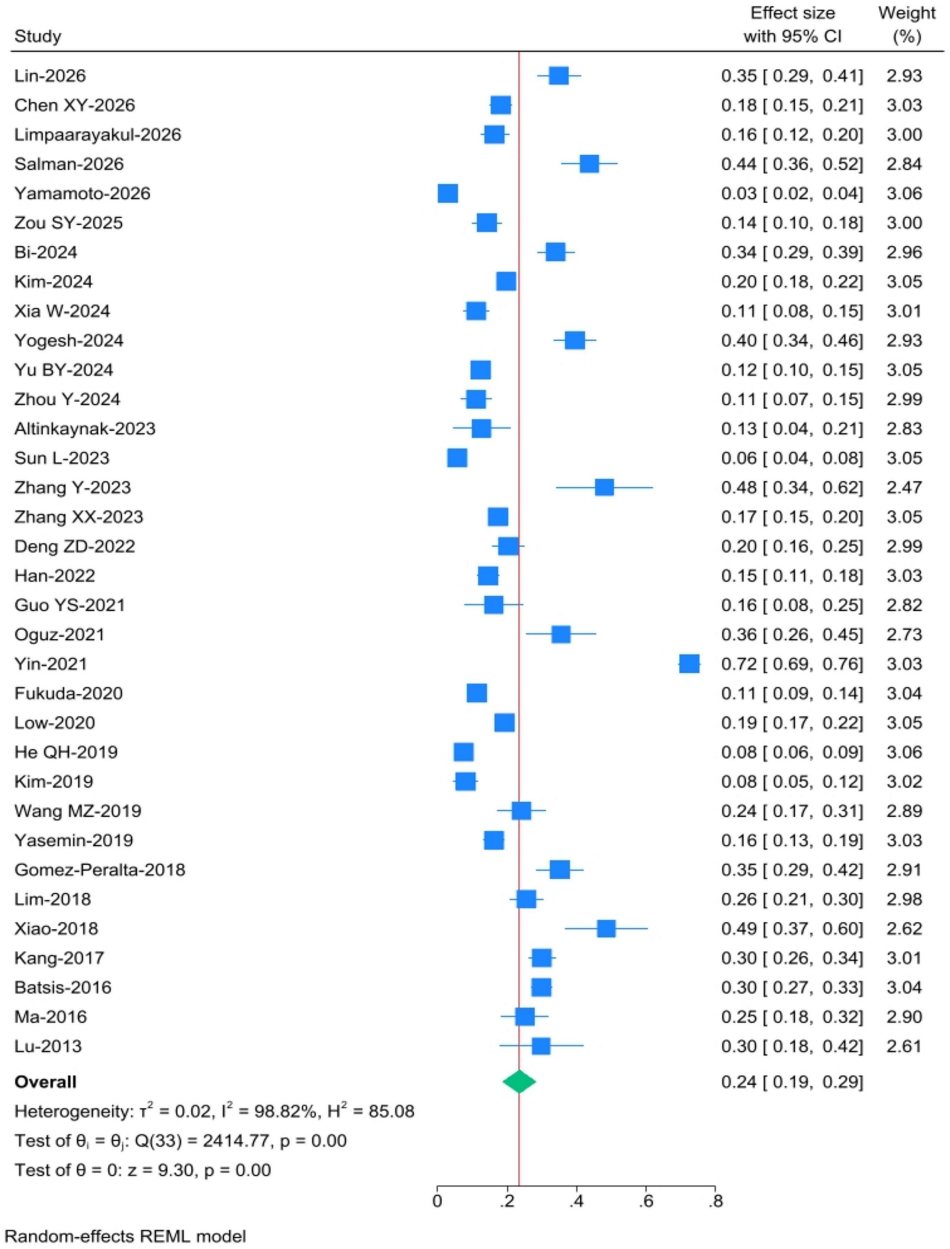
Forest plot of the prevalence of SO in adults with diabetes based on a random-effects model. SO, sarcopenic obesity; CI, confidence interval; REML, restricted maximum likelihood.

The prevalence of SO among adults with diabetes ranged from 3.0% (21) to 72.4% (46). Using a random-effects model, the pooled prevalence of SO among adults with diabetes was 24% (95% CI: 19–29%), with substantial between-study heterogeneity (*I^2^* = 98.82%, *p* < 0.001) (Figure 2). To further explore potential sources of heterogeneity, subgroup analyses were performed according to study setting, sex, continent, muscle mass assessment method, sarcopenia diagnostic criteria, and obesity definition (Table 2). By study setting, the pooled prevalence was highest in community-based studies (29, 95% CI: 18–40%; *I^2^* = 99.00%), followed by outpatient studies (24, 95% CI: 15–32%; *I^2^* = 99.04%) and inpatient studies (19, 95% CI: 12–25%; *I^2^* = 97.07%). Sex-specific subgroup analysis showed a higher pooled prevalence among female patients (28, 95% CI: 16–40%; *I^2^* = 99.45%) than among male patients (19, 95% CI: 10–28%; *I^2^* = 99.26%). Stratification by continent showed pooled prevalence estimates of 22% (95% CI: 17–28%; *I^2^* = 98.95%) in Asian populations and of 29% (95% CI: 17–42%; *I^2^* = 96.95%) in North American populations. Prevalence estimates also varied according to muscle mass assessment methods and diagnostic criteria. Studies using BIA reported a pooled prevalence of 25% (95% CI: 18–33%; *I^2^* = 99.10%), whereas studies using DXA reported a pooled prevalence of 23% (95% CI: 14–31%; *I^2^* = 98.32%). According to the SO criteria defined in Table 2, the pooled prevalence was 26% (95% CI: 19–33%; *I^2^* = 98.89%) for Method 1, 10% (95% CI: 6–14%; *I^2^* = 94.14%) for Method 2, and 27% (95% CI: 18–36%; *I^2^* = 96.70%) for Method 3. Regarding obesity definitions, the pooled prevalence was 22% (95% CI: 12–32%; *I^2^* = 99.20%) in studies using BFP, 25% (95% CI: 18–31%; *I^2^* = 93.84%) in studies using BMI, and 23% (95% CI: 12–35%; *I^2^* = 97.59%) in studies using WC.

**Table 2:** Heterogeneity analyses.

Heterogeneity analysis	Study	Heterogeneity		Effect model	ES (95% CI)
Subgroups					
		*I^2^* (%)	*P*		
Setting					
Outpatient	12	99.04	<0.001	Random	0.24 (0.15, 0.32)
Inpatient	11	97.07	<0.001	Random	0.19 (0.12, 0.25)
Community	10	99.00	<0.001	Random	0.29 (0.18, 0.40)
Gender					
Male	15	99.26	<0.001	Random	0.19 (0.10, 0.28)
Female	15	99.45	<0.001	Random	0.28 (0.16, 0.40)
Continent					
Asia	25	98.77	<0.001	Random	0.21 (0.16, 0.27)
North America	4	95.07	<0.001	Random	0.29 (0.19, 0.39)
Methods of muscle mass measurement					
BIA	20	99.10	<0.001	Random	0.25 (0.18, 0.33)
DXA	9	98.32	<0.001	Random	0.23 (0.14, 0.31)
Diagnostic criteria for sarcopenia					
Method 1	18	98.89	<0.001	Random	0.26 (0.19, 0.33)
Method 2	6	94.14	<0.001	Random	0.10 (0.06, 0.14)
Method 3	9	96.70	<0.001	Random	0.27 (0.18, 0.36)
Diagnostic criteria for obesity					
BFP	13	99.20	<0.001	Random	0.22 (0.12, 0.32)
BMI	9	93.84	<0.001	Random	0.25 (0.18, 0.31)
WC	5	97.59	<0.001	Random	0.23 (0.12, 0.35)

ASMI, appendicular skeletal muscle mass index; ASM, appendicular skeletal muscle mass; BFP, body fat percentage; BIA, bioelectrical impedance analysis; BMI, body mass index; CI, confidence interval; DXA, dual-energy X-ray absorptiometry; ES, effect size; SMI, skeletal muscle index; WC, waist circumference; Wt, weight. Method 1 assessed a single component of sarcopenia, either muscle mass or muscle strength. Muscle mass was represented by SMI, ASMI, or ASM / Wt, and muscle strength was assessed by handgrip strength. Method 2 assessed two of the three components, namely, muscle mass, muscle strength, and physical performance, with physical performance represented by gait speed. Method 3 assessed all three components, including muscle mass, muscle strength, and physical performance.

Substantial heterogeneity persisted across most subgroups, suggesting that differences in study setting, sex, geographic region, muscle mass assessment method, and diagnostic criteria may partly explain the variability in reported prevalence estimates. These findings indicate that the pooled prevalence should be interpreted with caution and highlight the importance of standardized assessment criteria for SO in adults with diabetes.

### 3.4 Factors associated with SO

A limited number of included studies reported factors associated with SO or SO-related body composition phenotypes among adults with diabetes; therefore, these factors were summarized descriptively because of differences in variable definitions, statistical models, adjustment factors, and effect measures. Overall, the reported factors were mainly related to body composition and adiposity, glycemic metabolism, anthropometric and functional indicators, and diabetes-related clinical characteristics. Body composition-related indicators, including body mass index, fat mass index, and limb fat mass, were reported in relation to SO-related low muscle mass or fat mass/fat-free mass (FM/FFM)-defined SO phenotypes (22, 30). Metabolic factors included low serum irisin levels of < 9.49 ng/mL and poorer glycemic control or higher glycated hemoglobin levels (20, 30). Anthropometric and functional indicators associated with SO included A Body Shape Index ≥ 0.083, older age, smaller calf circumference, and lower handgrip strength (19, 20, 36). In addition, low physical activity, insulin use, and diabetes-related complications were reported as diabetes-related or clinical factors associated with SO, whereas hypertension and dyslipidemia were not consistently associated with SO (20).

### 3.5 Sensitivity analyses

**Figure 3:**
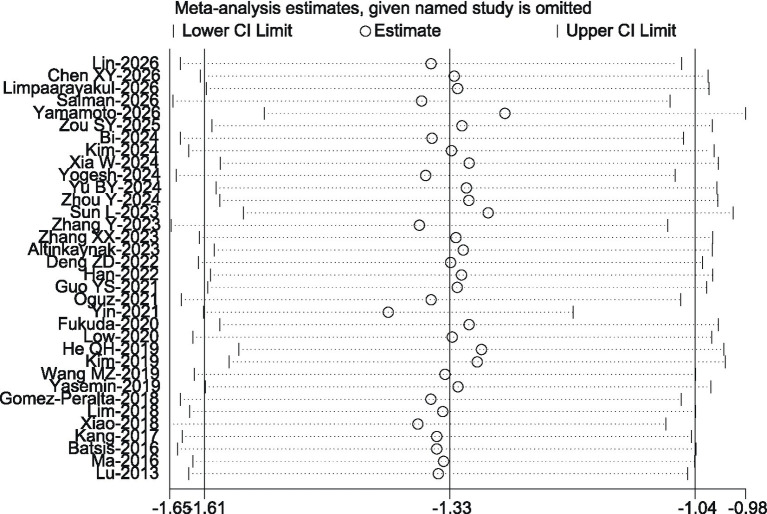
Sensitivity analysis for studies included in the meta-analysis. CI, confidence interval.

A leave-one-out sensitivity analysis was conducted by sequentially omitting each of the 34 included studies to evaluate the influence of individual studies on the pooled prevalence estimate. The pooled estimates changed only slightly after the omission of each study, indicating that the overall result was relatively stable and was not driven by any single study. Among the included studies, omission of Yin-2021 (46) produced the largest downward shift in the pooled estimate; however, this change did not materially alter the primary conclusion (Figure 3).

### 3.6 Publication bias

**Figure 4:**
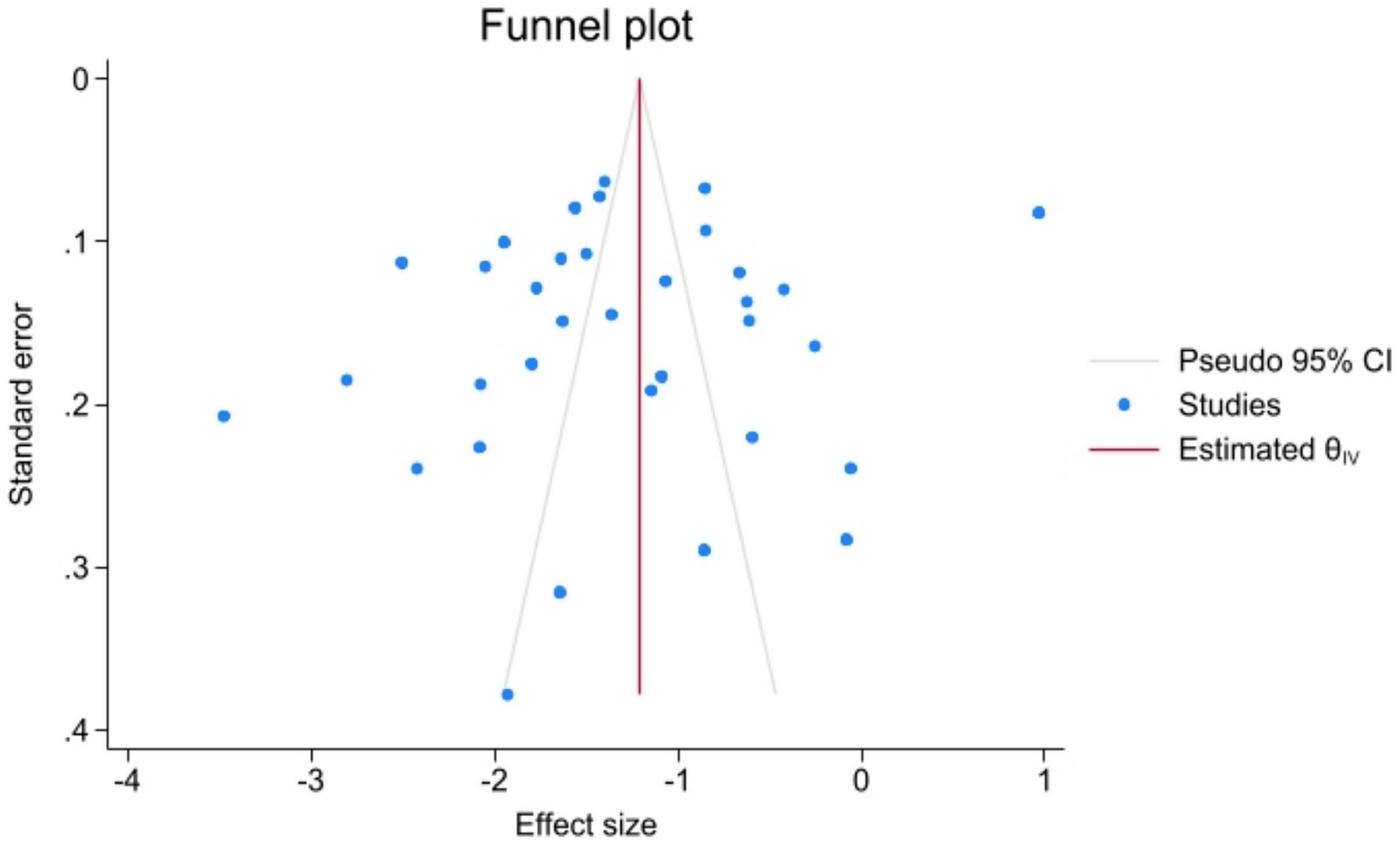
Funnel plot for studies included in the meta-analysis. CI, confidence interval.

Publication bias was assessed using funnel plot visualization and Egger’s regression test based on logit-transformed prevalence estimates (Figure 4). The funnel plot appeared approximately symmetrical around the pooled effect size, suggesting no obvious publication bias. Egger’s regression test showed no significant small-study effects (β1 = −1.34, 95% CI: −5.42 to 2.74, *p* = 0.518), indicating no statistical evidence of substantial publication bias.

### 3.7 Certainty of evidence

The certainty of evidence for the pooled prevalence of SO among adults with diabetes was rated as very low. This rating primarily reflected very substantial between-study heterogeneity and indirectness related to variability in study settings, populations, body composition assessment methods, and diagnostic criteria for SO rather than uniformly poor methodological quality of the included studies. Risk of bias was rated as serious because a majority of the studies were of moderate quality, and eight studies were rated as low quality according to the AHRQ checklist. Imprecision was not considered serious because the CI around the pooled prevalence estimate was acceptable. No serious publication bias was detected based on visual inspection of the funnel plot and Egger’s regression test. The detailed GRADE certainty assessment is provided in Supplementary Table 11.

## 4 Discussion

This systematic review and meta-analysis synthesized evidence from 34 observational studies published between 2013 and 2026 and estimated that the pooled prevalence of SO among adults with diabetes was 24%. This finding suggests that approximately one in four adults with diabetes may be affected by SO, indicating a substantial clinical and public health burden. The estimate is broadly consistent with the 27% reported by Zhou et al. (14). However, because between-study heterogeneity was extremely high, the pooled estimate should be interpreted with caution. Rather than representing a uniform prevalence derived from clinically and methodologically comparable studies, it should be regarded as an overall summary across heterogeneous populations, healthcare settings, body composition assessment methods, and diagnostic frameworks.

This heterogeneity is consistent with previous meta-analyses in related fields, in which substantial variability persisted after subgroup analyses or meta-regression (14, 51), suggesting that prevalence estimates of SO and sarcopenia in diabetic populations are unlikely to be explained by a single study-level factor. These findings indicate that heterogeneity in this field is likely multifactorial, reflecting the combined influence of population characteristics, study settings, assessment methods, and diagnostic criteria rather than a single dominant source.

Subgroup analyses suggested that study setting, sex distribution, and geographic region may partly contribute to the observed heterogeneity. The higher prevalence among female patients may be related to sex-specific differences in body composition, including greater adiposity and lower lean mass at comparable BMI levels, particularly after menopause, when estrogen decline may promote fat accumulation and impair muscle protein synthesis (52, 53). Geographic differences may reflect population-specific adiposity patterns, ethnic differences in body composition, lifestyle factors, healthcare settings, and diagnostic thresholds; however, this finding should be interpreted cautiously because the number of studies in some regions was limited (54). Prevalence also varied by study setting, with community-based studies showing a relatively higher pooled prevalence than outpatient and inpatient studies. This pattern may be related to differences in population selection, age structure, physical activity, nutritional status, diabetes duration, and access to assessment of body composition or muscle function (55, 56). Nevertheless, these subgroup findings should be interpreted as study-level patterns rather than causal explanations, because substantial heterogeneity persisted within most strata.

Body composition assessment methods may also influence the identification of SO. Prevalence estimates differed between studies using BIA and those using DXA. DXA is generally considered a more precise reference method for body composition assessment (57). BIA is simpler, less expensive, and more feasible in clinical and epidemiological settings, but its estimates can be affected by hydration status, device-specific equations, and population characteristics (58). Therefore, differences between BIA and DXA should be considered a methodological source of variation that may influence SO classification.

Variability in diagnostic criteria represents a more fundamental source of heterogeneity. SO is a composite phenotype, and variation may arise from both the sarcopenia and obesity components of the definition. Across the included studies, sarcopenia was defined using different combinations of muscle mass, muscle strength, and physical performance, reflecting differences among major consensus frameworks, including the International Working Group on Sarcopenia, the European Working Group on Sarcopenia in Older People, and the Asian Working Group for Sarcopenia (59–61). Some studies relied mainly on muscle mass-related indicators, whereas others incorporated handgrip strength, gait speed, the five-time sit-to-stand test, or the short physical performance battery. These approaches may capture different dimensions of sarcopenia, from reduced muscle quantity to impaired muscle function. The obesity component was also heterogeneous, with studies using BMI, waist circumference, percentage body fat, visceral fat area, fat mass to fat-free mass ratio, or composite criteria. These indicators are not interchangeable. BMI reflects general adiposity but cannot distinguish lean mass from fat mass; waist circumference and visceral fat area better reflect central or visceral adiposity; and percentage body fat captures overall fat accumulation but provides less information on fat distribution (62, 63). Therefore, studies using different diagnostic frameworks may identify different clinical phenotypes, limiting the direct comparability of prevalence estimates.

The study by Chen et al. (18) provides a clear example of how obesity criteria can alter both prevalence estimates and clinical interpretation within the same population. Among adults with type 2 diabetes, SO prevalence differed substantially when obesity was defined using BMI, percentage body fat, waist circumference, or visceral fat area. The association with fall risk also differed by definition, with SO defined by visceral fat area showing the strongest association. This example indicates that diagnostic criteria influence not only the numerical prevalence of SO but also the clinical risk profile captured by the diagnosis. Accordingly, the pooled prevalence in this meta-analysis should be interpreted as an overall estimate across heterogeneous diagnostic frameworks rather than as a fully comparable estimate across all included studies.

Recent evidence also supports the clinical relevance of SO screening in diabetes care. Yilmaz et al. (64) evaluated SO risk among diabetic outpatients using the SARC-F questionnaire, a five-item sarcopenia screening tool, and handgrip strength, suggesting that simple screening tools may help identify patients who require further assessment. This is particularly relevant in outpatient diabetes clinics, where advanced body composition methods such as DXA may not be routinely available. However, screening-based risk assessment should not be considered equivalent to standardized diagnostic classification. Patients with positive screening results may require confirmatory evaluation using validated body composition measures combined with muscle strength or physical performance assessments.

The associated factors summarized in this review suggest that SO in adults with diabetes is a multifactorial phenotype rather than the consequence of a single clinical pathway. Mechanistically, SO may arise from the interaction of altered body composition, abnormal fat distribution, impaired glucose metabolism, and dysfunctional adipose–muscle crosstalk, which can promote chronic low-grade inflammation, insulin resistance, impaired anabolic signaling, and increased proteolysis (65, 66). This biological framework is consistent with the observed clustering of factors related to adiposity, glycemic metabolism, muscle function, physical activity, and diabetes-related clinical burden. Body composition-related indicators, including BMI, fat mass index, limb fat mass, A Body Shape Index, and visceral adiposity, reflect different dimensions of adiposity and fat distribution, and their clinical meaning may vary according to how SO is defined (18, 22, 30, 36). These indicators should therefore be interpreted together with direct measures of muscle mass, muscle strength, and physical performance rather than as interchangeable markers of SO. Metabolic and functional factors, including poorer glycemic control, low serum irisin levels, low physical activity, smaller calf circumference, and lower handgrip strength, further suggest the relevance of chronic hyperglycemia, altered myokine signaling, reduced physical activity, and declining muscle reserve (19, 20, 30). Insulin use and diabetes-related complications may also indicate a greater diabetes-related clinical burden among patients with SO, although these associations should not be interpreted as causal. Since most available studies were cross-sectional and used heterogeneous definitions and statistical models, the reported factors should be interpreted as associated factors rather than causal determinants.

## 5 Conclusion

In conclusion, the findings of this systematic review and meta-analysis suggest that SO is common among adults with diabetes, with an estimated pooled prevalence of 24%. However, this estimate should be interpreted with caution because substantial heterogeneity was observed across the included studies. Differences in population characteristics, study settings, body composition assessment methods, and diagnostic criteria for both sarcopenia and obesity may have contributed to the variability in prevalence estimates and limited their direct comparability. These findings highlight the need for greater clinical attention to SO in adults with diabetes, particularly among individuals with reduced muscle reserve, increased adiposity, poor glycemic control, or impaired physical function. Future high-quality prospective studies using standardized measurement approaches and harmonized diagnostic criteria are needed to improve the accuracy, comparability, and clinical applicability of SO assessment in diabetes care.

### 5.1 Strengths, limitations, and future directions

This systematic review and meta-analysis have several strengths. First, it provides an updated and comprehensive synthesis of the available evidence on the prevalence of SO among adults with diabetes and further summarizes the reported factors associated with SO. Second, the literature search was conducted across both international and Chinese databases, which improved the breadth and sensitivity of evidence retrieval. Third, study selection, data extraction, and methodological quality assessment were performed independently by two reviewers, reducing the likelihood of selection and extraction bias. In addition, subgroup analyses, sensitivity analysis, a publication bias assessment, and a certainty of evidence assessment were performed to evaluate the stability and interpretability of the pooled findings.

Several limitations should also be acknowledged. First, substantial heterogeneity was observed across the included studies, which represents a major limitation of this meta-analysis. This heterogeneity may be partly attributable to differences in diagnostic criteria for SO, study settings, geographic regions, body composition assessment methods, and population characteristics. In particular, variation in the definitions of both sarcopenia and obesity limited the direct comparability of prevalence estimates across studies. Second, a majority of the included studies were observational and cross-sectional in design, which limited causal inference regarding factors associated with SO. Third, although factors associated with SO were summarized, the number of studies reporting these factors was limited, and the definitions, statistical models, and effect measures varied substantially across studies. Therefore, these factors could only be summarized descriptively rather than quantitatively pooled. Fourth, individual participant data were unavailable, which prevented more detailed stratified analyses by age, sex, diabetes duration, glycemic control, comorbidity burden, and diagnostic components. Finally, because conference abstracts and grey literature were excluded, some relevant evidence may have been missed.

Future research should prioritize high-quality prospective and multicenter studies to clarify the longitudinal associations between diabetes-related characteristics and SO and to explore potential causal pathways further. Studies should adopt standardized measurement approaches and harmonized diagnostic criteria, clearly report the sarcopenia and obesity components of the diagnosis, and provide prevalence estimates stratified by study setting, sex, age group, diabetes type, and body composition assessment method. In addition, future studies should use consistent definitions and effect measures when reporting associated factors, which would improve the feasibility of quantitative synthesis and enhance the comparability and clinical applicability of evidence in diabetes care.

## Data Availability

The original contributions presented in the study are included in the article/Supplementary material, further inquiries can be directed to the corresponding authors.
