# Supplementary material for: Prevalence of sarcopenic obesity in adult patients with diabetes and associated factors: a systematic review and meta-analysis
Source: Front Nutr. 2026 Jul 23;13:1880656. doi: 10.3389/fnut.2026.1880656 (PMC13442009; doi:10.3389/fnut.2026.1880656)
Supplement: Supplementary file 1 [file Table_1.docx]

Supplementary Material

**eTable 1. Completed PRISMA 2020 Checklist**

**eTable 2. Search Strategy: MEDLINE via PubMed**

**eTable 3. Search Strategy: Embase**

**eTable 4. Search Strategy: Cochrane Library**

**eTable 5. Search Strategy: SinoMed**

**eTable 6. Search Strategy: CNKI**

**eTable 7. Search Strategy: Wanfang Database**

**eTable 8. Search Strategy: VIP**

**eTable 9. Agency for Healthcare Research and Quality Checklist for Cross-sectional Studies**

**eTable 10. Methodological Quality Assessment of Included Studies Using the AHRQ Checklist**

**eTable 11. GRADE Certainty Assessment for the Pooled Prevalence of SO**

**eTable 1. Completed PRISMA 2020 Checklist**

A completed PRISMA 2020 checklist is provided to indicate where each reporting item is addressed in the manuscript and supplementary materials.

| **Section and Topic** | **Item #** | **Checklist item** | **Location where item is reported** |
| --- | --- | --- | --- |
| **TITLE** | | |  |
| Title | 1 | Identify the report as a systematic review. | Title page |
| **ABSTRACT** | | |  |
| Abstract | 2 | Structured abstract including objective, methods, results, and conclusion. | Abstract |
| **INTRODUCTION** | | |  |
| Rationale | 3 | Describe the rationale for the review in the context of existing knowledge. | Introduction, paragraphs 1-3 |
| Objectives | 4 | Provide an explicit statement of the objective(s) or question(s) the review addresses. | Introduction, final paragraph |
| **METHODS** | | |  |
| Eligibility criteria | 5 | Specify the inclusion and exclusion criteria for the review and how studies were grouped for the syntheses. | Section 2.2; Section 2.4; Section 2.6 |
| Information sources | 6 | Specify all databases, registers, websites, organisations, reference lists and other sources searched or consulted to identify studies. Specify the date when each source was last searched or consulted. | Section 2.1; Suppl. eTables 2-8 |
| Search strategy | 7 | Present the full search strategies for all databases, registers and websites, including any filters and limits used. | Section 2.1; Suppl. eTables 2-8 |
| Selection process | 8 | Specify the methods used to decide whether a study met the inclusion criteria of the review, including how many reviewers screened each record and each report retrieved, whether they worked independently, and if applicable, details of automation tools used in the process. | Section 2.3 |
| Data collection process | 9 | Specify the methods used to collect data from reports, including how many reviewers collected data from each report, whether they worked independently, any processes for obtaining or confirming data from study investigators, and if applicable, details of automation tools used in the process. | Section 2.4 |
| Data items | 10a | List and define all outcomes for which data were sought. Specify whether all results that were compatible with each outcome domain in each study were sought (e.g. for all measures, time points, analyses), and if not, the methods used to decide which results to collect. | Section 2.4; Section 2.6 |
|  | 10b | List and define all other variables for which data were sought (e.g. participant and intervention characteristics, funding sources). Describe any assumptions made about any missing or unclear information. | Section 2.4; Table 1 |
| Study risk of bias assessment | 11 | Specify the methods used to assess risk of bias in the included studies, including details of the tool(s) used, how many reviewers assessed each study and whether they worked independently, and if applicable, details of automation tools used in the process. | Section 2.5; Suppl. eTable 9 |
| Effect measures | 12 | Specify for each outcome the effect measure(s) (e.g. risk ratio, mean difference) used in the synthesis or presentation of results. | Section 2.6, pooled prevalence with 95% CI |
| Synthesis methods | 13a | Describe the processes used to decide which studies were eligible for each synthesis (e.g. tabulating the study intervention characteristics and comparing against the planned groups for each synthesis (item #5)). | Sections 2.2, 2.4 and 2.6 |
|  | 13b | Describe any methods required to prepare the data for presentation or synthesis, such as handling of missing summary statistics, or data conversions. | Section 2.4 |
|  | 13c | Describe any methods used to tabulate or visually display results of individual studies and syntheses. | Section 2.6; Tables 1-2; Figures 2-4 |
|  | 13d | Describe any methods used to synthesize results and provide a rationale for the choice(s). If meta-analysis was performed, describe the model(s), method(s) to identify the presence and extent of statistical heterogeneity, and software package(s) used. | Section 2.6 |
|  | 13e | Describe any methods used to explore possible causes of heterogeneity among study results (e.g. subgroup analysis, meta-regression). | Section 2.6 |
|  | 13f | Describe any sensitivity analyses conducted to assess robustness of the synthesized results. | Section 2.6 |
| Reporting bias assessment | 14 | Describe any methods used to assess risk of bias due to missing results in a synthesis (arising from reporting biases). | Section 2.6 |
| Certainty assessment | 15 | Describe any methods used to assess certainty (or confidence) in the body of evidence for an outcome. | Section 2.7; Suppl. eTable 11 |
| **RESULTS** | | |  |
| Study selection | 16a | Describe the results of the search and selection process, from the number of records identified in the search to the number of studies included in the review, ideally using a flow diagram. | Section 3.1; Figure 1 |
|  | 16b | Cite studies that might appear to meet the inclusion criteria, but which were excluded, and explain why they were excluded. | Section 3.1; Figure 1, full-text exclusions with reasons |
| Study characteristics | 17 | Cite each included study and present its characteristics. | Section 3.2; Table 1; Refs. [17-50] |
| Risk of bias in studies | 18 | Present assessments of risk of bias for each included study. | Section 3.2; Table 1; Suppl. eTable 10 |
| Results of individual studies | 19 | For all outcomes, present, for each study: (a) summary statistics for each group (where appropriate) and (b) an effect estimate and its precision (e.g. confidence/credible interval), ideally using structured tables or plots. | Sections 3.2-3.4; Table 1; Figure 2 |
| Results of syntheses | 20a | For each synthesis, briefly summarise the characteristics and risk of bias among contributing studies. | Section 3.2; Table 1; Suppl. eTable 10 |
|  | 20b | Present results of all statistical syntheses conducted. If meta-analysis was done, present for each the summary estimate and its precision (e.g. confidence/credible interval) and measures of statistical heterogeneity. If comparing groups, describe the direction of the effect. | Section 3.3; Figure 2; Table 2 |
|  | 20c | Present results of all investigations of possible causes of heterogeneity among study results. | Section 3.3; Table 2 |
|  | 20d | Present results of all sensitivity analyses conducted to assess the robustness of the synthesized results. | Section 3.5; Figure 3 |
| Reporting biases | 21 | Present assessments of risk of bias due to missing results (arising from reporting biases) for each synthesis assessed. | Section 3.6; Figure 4 |
| Certainty of evidence | 22 | Present assessments of certainty (or confidence) in the body of evidence for each outcome assessed. | Section 3.7; Suppl. eTable 11 |
| **DISCUSSION** | | |  |
| Discussion | 23a | Provide a general interpretation of the results in the context of other evidence. | Discussion section |
|  | 23b | Discuss any limitations of the evidence included in the review. | Section 5.1, limitations paragraph |
|  | 23c | Discuss any limitations of the review processes used. | Section 5.1, limitations paragraph |
|  | 23d | Discuss implications of the results for practice, policy, and future research. | Section 5; Section 5.1, final paragraph |
| **OTHER INFORMATION** | | |  |
| Registration and protocol | 24a | Provide registration information for the review, including register name and registration number, or state that the review was not registered. | Section 2 Materials and methods; PROSPERO registration |
|  | 24b | Indicate where the review protocol can be accessed, or state that a protocol was not prepared. | Section 2 Materials and methods; PROSPERO registration record CRD420251274126 |
|  | 24c | Describe and explain any amendments to information provided at registration or in the protocol. | No protocol amendments were reported |
| Support | 25 | Describe sources of financial or non-financial support for the review, and the role of the funders or sponsors in the review. | Funding |
| Competing interests | 26 | Declare any competing interests of review authors. | Conflict of interest |
| Availability of data, code and other materials | 27 | Report which of the following are publicly available and where they can be found: template data collection forms; data extracted from included studies; data used for all analyses; analytic code; any other materials used in the review. | Data availability statement; Table 1; Supplementary Material |

**eTable 2. Search Strategy: MEDLINE via PubMed**

| #1 | ''Diabetes Mellitus''[MeSH Terms] OR ''Diabetes Complications''[MeSH Terms] OR ''Hyperglycemia''[MeSH Terms] OR ''Glucose Intolerance''[MeSH Terms] |
| --- | --- |
| #2 | ''Diabetes Mellitus''[Title/Abstract] OR ''Diabetes Insipidus''[Title/Abstract] OR ''Diet, Diabetic''[Title/Abstract] OR ''Prediabetic State''[Title/Abstract] OR ''Scleredema Adultorum''[Title/Abstract] OR ''Glycation End Products, Advanced''[Title/Abstract] OR ''Glucose Intolerance''[Title/Abstract] OR ''Gastroparesis''[Title/Abstract] OR ''Diabetes Complications''[Title/Abstract] OR ''Diabetes Complication''[Title/Abstract] OR ''Diabetic Complications''[Title/Abstract] OR ''Diabetic Complication''[Title/Abstract] OR ''Complications of Diabetes Mellitus''[Title/Abstract] OR ''Diabetes Mellitus Complication''[Title/Abstract] OR ''Diabetes Mellitus Complications''[Title/Abstract] OR ''Diabetes-Related Complications''[Title/Abstract] OR ''Diabetes-Related Complication''[Title/Abstract] OR ''Diabetes Related Complications''[Title/Abstract] OR Hyperglycemia[Title/Abstract] OR ''Hyperglycemias''[Title/Abstract] OR ''Hyperglycemia, Postprandial''[Title/Abstract] OR ''Hyperglycemias, Postprandial''[Title/Abstract] OR ''Postprandial Hyperglycemias''[Title/Abstract] OR ''Postprandial Hyperglycemia''[Title/Abstract] OR ''Glucose Intolerance''[Title/Abstract] OR ''Glucose Intolerances''[Title/Abstract] OR ''Intolerance, Glucose''[Title/Abstract] OR ''Intolerances, Glucose''[Title/Abstract] OR ''Impaired Glucose Tolerance''[Title/Abstract] OR ''Glucose Tolerance, Impaired''[Title/Abstract] OR ''Glucose Tolerances, Impaired''[Title/Abstract] OR ''Impaired Glucose Tolerances''[Title/Abstract] OR ''Tolerance, Impaired Glucose''[Title/Abstract] OR ''Tolerances, Impaired Glucose''[Title/Abstract] OR ''Impaired fasting glucose''[Title/Abstract] OR ''Impaired glucose tolerance''[Title/Abstract] |
| #3 | ''Obesity''[MeSH Terms] OR ''Overweight''[MeSH Terms] OR ''Obesity, Abdominal''[MeSH Terms] OR ''Body Composition''[MeSH Terms] OR ''Weight Loss''[MeSH Terms] OR ''Body Weight''[MeSH Terms] OR ''Body Mass Index''[MeSH Terms] |
| #4 | ''Obesity''[Title/Abstract] OR ''Overweight''[Title/Abstract] OR ''Obesity, Abdominal''[Title/Abstract] OR ''Abdominal Obesities''[Title/Abstract] OR ''Obesities, Abdominal''[Title/Abstract] OR ''Abdominal Obesity''[Title/Abstract] OR ''Central Obesity''[Title/Abstract] OR ''Central Obesities''[Title/Abstract] OR ''Obesities, Central''[Title/Abstract] OR ''Obesity, Central''[Title/Abstract] OR ''Obesity, Visceral''[Title/Abstract] OR ''Visceral Obesity''[Title/Abstract] OR ''Obesities, Visceral''[Title/Abstract] OR ''Visceral Obesities''[Title/Abstract] OR ''Body Composition''[Title/Abstract] OR ''Fat accumulation''[Title/Abstract] OR ''Body Compositions''[Title/Abstract] OR ''Composition, Body''[Title/Abstract] OR ''Compositions, Body''[Title/Abstract] OR ''Weight Loss''[Title/Abstract] OR ''Losses, Weight''[Title/Abstract] OR ''Loss, Weight''[Title/Abstract] OR ''Weight Losses''[Title/Abstract] OR ''Weight Reduction''[Title/Abstract] OR ''Reductions, Weight''[Title/Abstract] OR ''Reduction, Weight''[Title/Abstract] OR ''Weight Reductions''[Title/Abstract] OR ''Body Weigh''[Title/Abstract] OR ''Body Weights''[Title/Abstract] OR ''Weight, Body''[Title/Abstract] OR ''Weights, Body''[Title/Abstract] OR ''Body Mass Index''[Title/Abstract] OR ''Index, Body Mass''[Title/Abstract] OR ''Quetelet's Index''[Title/Abstract] OR ''Quetelets Index''[Title/Abstract] OR ''Quetelet Index''[Title/Abstract] OR ''Index, Quetelet''[Title/Abstract] OR ''BMI''[Title/Abstract] |
| #5 | ''Sarcopenia''[MeSH Terms] OR ''Muscle Weakness''[MeSH Terms] OR ''Muscular Atrophy''[MeSH Terms] OR ''Muscle, Skeletal''[MeSH Terms] |
| #6 | ''Sarcopenia''[Title/Abstract] OR ''Sarcopenias''[Title/Abstract] OR ''Muscle Weakness''[Title/Abstract] OR ''Muscle Weaknesses''[Title/Abstract] OR ''Weaknesses, Muscle''[Title/Abstract] OR ''Weakness, Muscle''[Title/Abstract] OR ''Musular Weakness''[Title/Abstract] OR ''Musular Weaknesses''[Title/Abstract] OR ''Weakneses, Muscular''[Title/Abstract] OR ''Weakness, Muscular''[Title/Abstract] OR ''Muscular Atrophy''[Title/Abstract] OR ''Atrophies, Muscular''[Title/Abstract] OR ''Atrophy, Muscular''[Title/Abstract] OR ''Muscular Atrophies''[Title/Abstract] OR ''Atrophy, Muscle''[Title/Abstract] OR ''Atrophies, Muscle''[Title/Abstract] OR ''Muscle Atrophies''[Title/Abstract] OR ''Muscle Atrophy''[Title/Abstract] OR ''Neurogenic Muscular Atrophy''[Title/Abstract] OR ''Atrophies, Neurogenic Muscular''[Title/Abstract] OR ''Atrophy, Neurogenic Muscular''[Title/Abstract] OR ''Muscular Atrophies, Neurogenic''[Title/Abstract] OR ''Muscular Atrophy, Neurogenic''[Title/Abstract] OR ''Neurogenic Muscular Atrophies''[Title/Abstract] OR ''Neurotrophic Muscular Atrophy''[Title/Abstract] OR ''Atrophies, Neurotrophic Muscular''[Title/Abstract] OR ''Atrophy, Neurotrophic Muscular''[Title/Abstract] OR ''Muscular Atrophies, Neurotrophic''[Title/Abstract] OR ''Muscular Atrophy, Neurotrophic''[Title/Abstract] OR ''Neurotrophic Muscular Atrophies''[Title/Abstract] OR ''Muscle, Skeletal''[Title/Abstract] OR ''Mucles, Skeletal''[Title/Abstract] OR ''Skeletal Muscles''[Title/Abstract] OR ''Skeletal Muscle''[Title/Abstract] OR ''Muscle, Voluntary''[Title/Abstract] OR ''Muscles, Voluntary''[Title/Abstract] OR ''Voluntary Muscle''[Title/Abstract] OR ''Voluntary Muscles''[Title/Abstract] OR ''Anterior Tibial Muscle''[Title/Abstract] OR ''Muscle, Anterior Tibial''[Title/Abstract] OR ''Tibial Muscle, Anterior''[Title/Abstract] OR ''Gastrocnemius Muscle''[Title/Abstract] OR ''Muscle, Gastrocnemius''[Title/Abstract] OR ''Plantaris Muscle''[Title/Abstract] OR ''Muscle, Plantaris''[Title/Abstract] OR ''Soleus Muscle''[Title/Abstract] OR ''Muscle, Soleus''[Title/Abstract] OR ''Muscle wasting''[Title/Abstract] OR ''Muscle mass''[Title/Abstract] OR ''Muscle loss''[Title/Abstract] |
| #7 | #1 OR #2 |
| #8 | #3 OR #4 |
| #9 | #5 OR #6 |
| #10 | (#8 AND #9 OR ''Sarcopenia Obesity''[Title/Abstract]) AND #7 |

**eTable 3. Search Strategy: Embase**

| #1 | 'Diabetes Mellitus'/exp OR 'Diabetes Complications'/exp OR 'Hyperglycemia'/exp OR 'Glucose Intolerance'/exp |
| --- | --- |
| #2 | 'Diabetes Mellitus':ab,ti OR 'Diabetes Insipidus':ab,ti OR 'Diet, Diabetic':ab,ti OR 'Prediabetic State':ab,ti OR 'Scleredema Adultorum':ab,ti OR 'Glycation End Products, Advanced':ab,ti OR 'Glucose Intolerance':ab,ti OR 'Gastroparesis':ab,ti OR 'Diabetes Complications':ab,ti OR 'Diabetes Complication':ab,ti OR 'Diabetic Complications':ab,ti OR 'Diabetic Complication':ab,ti OR 'Complications of Diabetes Mellitus':ab,ti OR 'Diabetes Mellitus Complication':ab,ti OR 'Diabetes Mellitus Complications':ab,ti OR 'Diabetes-Related Complications':ab,ti OR 'Diabetes-Related Complication':ab,ti OR 'Diabetes Related Complications':ab,ti OR 'Hyperglycemia':ab,ti OR 'Hyperglycemias':ab,ti OR 'Hyperglycemia, Postprandial':ab,ti OR 'Hyperglycemias, Postprandial':ab,ti OR 'Postprandial Hyperglycemias':ab,ti OR 'Postprandial Hyperglycemia':ab,ti OR 'Glucose Intolerance':ab,ti OR 'Glucose Intolerances':ab,ti OR 'Intolerance, Glucose':ab,ti OR 'Intolerances, Glucose':ab,ti OR 'Impaired Glucose Tolerance':ab,ti OR 'Glucose Tolerance, Impaired':ab,ti OR 'Glucose Tolerances, Impaired':ab,ti OR 'Impaired Glucose Tolerances':ab,ti OR 'Tolerance, Impaired Glucose':ab,ti OR 'Tolerances, Impaired Glucose':ab,ti OR 'Impaired fasting glucose':ab,ti OR 'Impaired glucose tolerance':ab,ti |
| #3 | 'Obesity'/exp OR 'Overweight'/exp OR 'Obesity, Abdominal'/exp OR 'Body Composition'/exp OR 'Weight Loss'/exp OR 'Body Weight'/exp OR 'Body Mass Index'/exp |
| #4 | 'Obesity':ab,ti OR 'Overweight':ab,ti OR 'Obesity, Abdominal':ab,ti OR 'Abdominal Obesities':ab,ti OR 'Obesities, Abdominal':ab,ti OR 'Abdominal Obesity':ab,ti OR 'Central Obesity':ab,ti OR 'Central Obesities':ab,ti OR 'Obesities, Central':ab,ti OR 'Obesity, Central':ab,ti OR 'Obesity, Visceral':ab,ti OR 'Visceral Obesity':ab,ti OR 'Obesities, Visceral':ab,ti OR 'Visceral Obesities':ab,ti OR 'Body Composition':ab,ti OR 'Fat accumulation':ab,ti OR 'Body Compositions':ab,ti OR 'Composition, Body':ab,ti OR 'Compositions, Body':ab,ti OR 'Weight Loss':ab,ti OR 'Losses, Weight':ab,ti OR 'Loss, Weight':ab,ti OR 'Weight Losses':ab,ti OR 'Weight Reduction':ab,ti OR 'Reductions, Weight':ab,ti OR 'Reduction, Weight':ab,ti OR 'Weight Reductions':ab,ti OR 'Body Weight':ab,ti OR 'Body Weights':ab,ti OR 'Weight, Body':ab,ti OR 'Weights, Body':ab,ti OR 'Body Mass Index':ab,ti OR 'Index, Body Mass':ab,ti OR 'Quetelets Index':ab,ti OR 'Quetelet Index':ab,ti OR 'Index, Quetelet':ab,ti OR 'BMI':ab,ti |
| #5 | 'Sarcopenia'/exp OR 'Muscle Weakness'/exp OR 'Muscular Atrophy'/exp OR 'Muscle, Skeletal'/exp |
| #6 | 'Sarcopenia':ab,ti OR 'Sarcopenias':ab,ti OR 'Muscle Weakness':ab,ti OR 'Muscle Weaknesses':ab,ti OR 'Weaknesses, Muscle':ab,ti OR 'Weakness, Muscle':ab,ti OR 'Musular Weakness':ab,ti OR 'Musular Weaknesses':ab,ti OR 'Weakneses, Muscular':ab,ti OR 'Weakness, Muscular':ab,ti OR 'Muscular Atrophy':ab,ti OR 'Atrophies, Muscular':ab,ti OR 'Atrophy, Muscular':ab,ti OR 'Muscular Atrophies':ab,ti OR 'Atrophy, Muscle':ab,ti OR 'Atrophies, Muscle':ab,ti OR 'Muscle Atrophies':ab,ti OR 'Muscle Atrophy':ab,ti OR 'Neurogenic Muscular Atrophy':ab,ti OR 'Atrophies, Neurogenic Muscular':ab,ti OR 'Atrophy, Neurogenic Muscular':ab,ti OR 'Muscular Atrophies, Neurogenic':ab,ti OR 'Muscular Atrophy, Neurogenic':ab,ti OR 'Neurogenic Muscular Atrophies':ab,ti OR 'Neurotrophic Muscular Atrophy':ab,ti OR 'Atrophies, Neurotrophic Muscular':ab,ti OR 'Atrophy, Neurotrophic Muscular':ab,ti OR 'Muscular Atrophies, Neurotrophic':ab,ti OR 'Muscular Atrophy, Neurotrophic':ab,ti OR 'Neurotrophic Muscular Atrophies':ab,ti OR 'Muscle, Skeletal':ab,ti OR 'Mucles, Skeletal':ab,ti OR 'Skeletal Muscles':ab,ti OR 'Skeletal Muscle':ab,ti OR 'Muscle, Voluntary':ab,ti OR 'Muscles, Voluntary':ab,ti OR 'Voluntary Muscle':ab,ti OR 'Voluntary Muscles':ab,ti OR 'Anterior Tibial Muscle':ab,ti OR 'Muscle, Anterior Tibial':ab,ti OR 'Tibial Muscle, Anterior':ab,ti OR 'Gastrocnemius Muscle':ab,ti OR 'Muscle, Gastrocnemius':ab,ti OR 'Plantaris Muscle':ab,ti OR 'Muscle, Plantaris':ab,ti OR 'Soleus Muscle':ab,ti OR 'Muscle, Soleus':ab,ti OR 'Muscle wasting':ab,ti OR 'Muscle mass':ab,ti OR 'Muscle loss':ab,ti |
| #7 | #1 OR #2 |
| #8 | #3 OR #4 |
| #9 | #5 OR #6 |
| #10 | (#8 AND #9 OR 'Sarcopenia Obesity':ab,ti) AND #7 |

**eTable 4. Search Strategy: Cochrane Library**

| #1 | MeSH descriptor: [Diabetes Mellitus] explode all trees |
| --- | --- |
| #2 | MeSH descriptor: [Diabetes Complications] explode all trees |
| #3 | MeSH descriptor: [Hyperglycemia] explode all trees |
| #4 | MeSH descriptor: [Glucose Intolerance] explode all trees |
| #5 | (Diabetes Mellitus):ti,ab,kw OR (Diabetes Insipidus):ti,ab,kw OR (Diet, Diabetic):ti,ab,kw OR (Prediabetic State):ti,ab,kw OR (Scleredema Adultorum):ti,ab,kw OR (Glycation End Products, Advanced):ti,ab,kw OR (Glucose Intolerance):ti,ab,kw OR (Gastroparesis):ti,ab,kw OR (Diabetes Complications):ti,ab,kw OR (Diabetes Complication):ti,ab,kw OR (Diabetic Complications):ti,ab,kw OR (Diabetic Complication):ti,ab,kw OR (Complications of Diabetes Mellitus):ti,ab,kw OR (Diabetes Mellitus Complication):ti,ab,kw OR (Diabetes Mellitus Complications):ti,ab,kw OR (Diabetes-Related Complications):ti,ab,kw OR (Diabetes-Related Complication):ti,ab,kw OR (Diabetes Related Complications):ti,ab,kw OR (Hyperglycemia):ti,ab,kw OR (Hyperglycemias):ti,ab,kw OR (Hyperglycemia, Postprandial):ti,ab,kw OR (Hyperglycemias, Postprandial):ti,ab,kw OR (Postprandial Hyperglycemias):ti,ab,kw OR (Postprandial Hyperglycemia):ti,ab,kw OR (Glucose Intolerance):ti,ab,kw OR (Glucose Intolerances):ti,ab,kw OR (Intolerance, Glucose):ti,ab,kw OR (Intolerances, Glucose):ti,ab,kw OR (Impaired Glucose Tolerance):ti,ab,kw OR (Glucose Tolerance, Impaired):ti,ab,kw OR (Glucose Tolerances, Impaired):ti,ab,kw OR (Impaired Glucose Tolerances):ti,ab,kw OR (Tolerance, Impaired Glucose):ti,ab,kw OR (Tolerances, Impaired Glucose):ti,ab,kw OR (Impaired fasting glucose):ti,ab,kw OR (Impaired glucose tolerance):ti,ab,kw |
| #6 | MeSH descriptor: [Obesity] explode all trees |
| #7 | MeSH descriptor: [Overweight] explode all trees |
| #8 | MeSH descriptor: [Obesity, Abdominal] explode all trees |
| #9 | MeSH descriptor: [Body Composition] explode all trees |
| #10 | MeSH descriptor: [Weight Loss] explode all trees |
| #11 | MeSH descriptor: [Body Weight] explode all trees |
| #12 | MeSH descriptor: [Body Mass Index] explode all trees |
| #13 | (Obesity):ti,ab,kw OR (Overweight):ti,ab,kw OR (Obesity, Abdominal):ti,ab,kw OR (Abdominal Obesities):ti,ab,kw OR (Obesities, Abdominal):ti,ab,kw OR (Abdominal Obesity):ti,ab,kw OR (Central Obesity):ti,ab,kw OR (Central Obesities):ti,ab,kw OR (Obesities, Central):ti,ab,kw OR (Obesity, Central):ti,ab,kw OR (Obesity, Visceral):ti,ab,kw OR (Visceral Obesity):ti,ab,kw OR (Obesities, Visceral):ti,ab,kw OR (Visceral Obesities):ti,ab,kw OR (Body Composition):ti,ab,kw OR (Fat accumulation):ti,ab,kw OR (Body Compositions):ti,ab,kw OR (Composition, Body):ti,ab,kw OR (Compositions, Body):ti,ab,kw OR (Weight Loss):ti,ab,kw OR (Losses, Weight):ti,ab,kw OR (Loss, Weight):ti,ab,kw OR (Weight Losses):ti,ab,kw OR (Weight Reduction):ti,ab,kw OR (Reductions, Weight):ti,ab,kw OR (Reduction, Weight):ti,ab,kw OR (Weight Reductions):ti,ab,kw OR (Body Weight):ti,ab,kw OR (Body Weights):ti,ab,kw OR (Weight, Body):ti,ab,kw OR (Weights, Body):ti,ab,kw OR (Body Mass Index):ti,ab,kw OR (Index, Body Mass):ti,ab,kw OR (Quetelet's Index):ti,ab,kw OR (Quetelets Index):ti,ab,kw OR (Quetelet Index):ti,ab,kw OR (Index, Quetelet):ti,ab,kw OR (BMI):ti,ab,kw |
| #14 | MeSH descriptor: [Sarcopenia] explode all trees |
| #15 | MeSH descriptor: [Muscle Weakness] explode all trees |
| #16 | MeSH descriptor: [Muscular Atrophy] explode all trees |
| #17 | MeSH descriptor: [Muscle, Skeletal] explode all trees |
| #18 | (Sarcopenia):ti,ab,kw OR (Sarcopenias):ti,ab,kw OR (Muscle Weakness):ti,ab,kw OR (Muscle Weaknesses):ti,ab,kw OR (Weaknesses, Muscle):ti,ab,kw OR (Weakness, Muscle):ti,ab,kw OR (Musular Weakness):ti,ab,kw OR (Musular Weaknesses):ti,ab,kw OR (Weakneses, Muscular):ti,ab,kw OR (Weakness, Muscular):ti,ab,kw OR (Muscular Atrophy):ti,ab,kw OR (Atrophies, Muscular):ti,ab,kw OR (Atrophy, Muscular):ti,ab,kw OR (Muscular Atrophies):ti,ab,kw OR (Atrophy, Muscle):ti,ab,kw OR (Atrophies, Muscle):ti,ab,kw OR (Muscle Atrophies):ti,ab,kw OR (Muscle Atrophy):ti,ab,kw OR (Neurogenic Muscular Atrophy):ti,ab,kw OR (Atrophies, Neurogenic Muscular):ti,ab,kw OR (Atrophy, Neurogenic Muscular):ti,ab,kw OR (Muscular Atrophies, Neurogenic):ti,ab,kw OR (Muscular Atrophy, Neurogenic):ti,ab,kw OR (Neurogenic Muscular Atrophies):ti,ab,kw OR (Neurotrophic Muscular Atrophy):ti,ab,kw OR (Atrophies, Neurotrophic Muscular):ti,ab,kw OR (Atrophy, Neurotrophic Muscular):ti,ab,kw OR (Muscular Atrophies, Neurotrophic):ti,ab,kw OR (Muscular Atrophy, Neurotrophic):ti,ab,kw OR (Neurotrophic Muscular Atrophies):ti,ab,kw OR (Muscle, Skeletal):ti,ab,kw OR (Mucles, Skeletal):ti,ab,kw OR (Skeletal Muscles):ti,ab,kw OR (Skeletal Muscle):ti,ab,kw OR (Muscle, Voluntary):ti,ab,kw OR (Muscles, Voluntary):ti,ab,kw OR (Voluntary Muscle):ti,ab,kw OR (Voluntary Muscles):ti,ab,kw OR (Anterior Tibial Muscle):ti,ab,kw OR (Muscle, Anterior Tibial):ti,ab,kw OR (Tibial Muscle, Anterior):ti,ab,kw OR (Gastrocnemius Muscle):ti,ab,kw OR (Muscle, Gastrocnemius):ti,ab,kw OR (Plantaris Muscle):ti,ab,kw OR (Muscle, Plantaris):ti,ab,kw OR (Soleus Muscle):ti,ab,kw OR (Muscle, Soleus):ti,ab,kw OR (Muscle wasting):ti,ab,kw OR (Muscle mass):ti,ab,kw OR (Muscle loss):ti,ab,kw |
| #19 | #1 OR #2 OR #3 OR #4 OR #5 |
| #20 | #6 OR #7 OR #8 OR #9 OR #10 OR #11 OR #12 OR #13 |
| #21 | #14 OR #15 OR #16 OR #17 OR #18 |
| #22 | (#20 AND #21 OR (Sarcopenia Obesity):ti,ab,kw) AND #19 |

**eTable 5. Search Strategy: SinoMed**

| #1 | "肌肉衰减症"[常用字段] OR "肌少症"[常用字段] OR "少肌症"[常用字段] OR "肌肉萎缩"[常用字段] OR "肌肉减少症"[常用字段] （Muscular atrophy [Common field] OR Sarcopenia [Common field] OR Hypotonia [Common field] OR Muscle atrophy [Common field] OR Myohypoplasia [Common field]） |
| --- | --- |
| #2 | "肥胖"[常用字段] OR "超重"[常用字段] （"Obesity"[Common Field] OR "Overweight"[Common Field]） |
| #3 | "糖尿病"[常用字段] （Diabetes Mellitus [Common field]） |
| #4 | "少肌性肥胖症"[常用字段] OR "肌少性肥胖症"[常用字段] （Sarcopenic obesity [Common field] OR Skeletal Muscle Loss Obesity[Common field]） |
| #5 | #1 and #2 |
| #6 | #5 OR #4 |
| #7 | #6 AND #3 |

**eTable 6. Search Strategy: CNKI**

| #1 | SU=肌肉衰减症 OR SU=肌少症 OR SU=少肌症 OR SU=肌肉萎缩 OR SU=肌肉减少症 （SU = muscle atrophy disorder; OR SU = sarcopenia; OR SU = hypotonia; OR SU = muscle wasting; OR SU = muscle reduction syndrome） |
| --- | --- |
| #2 | SU=肥胖 OR SU=超重 （SU = Obesity OR SU = Overweight） |
| #3 | SU=糖尿病 （SU=Diabetes Mellitus） |
| #4 | SU=少肌性肥胖症 OR SU=肌少性肥胖症 （SU=Sarcopenic Obesity OR SU=Skeletal Muscle Loss Obesity） |
| #5 | #1 AND #2 |
| #6 | #5 OR #4 |
| #7 | #6 AND #3 |

**eTable 7. Search Strategy: Wanfang Database**

| #1 | 主题= 肌肉衰减症 OR 肌少症 OR 少肌症 OR 肌肉萎缩 OR 肌肉减少症 （Subject= muscle atrophy disorder OR sarcopenia OR hypotonia OR muscle wasting OR muscle reduction syndrome） |
| --- | --- |
| #2 | 主题= 肥胖 or 超重 （Subject= Obesity OR Overweight） |
| #3 | 主题= 糖尿病 （Subject= Diabetes Mellitus） |
| #4 | 主题= 少肌性肥胖症 OR 肌少性肥胖症 （Subject= Sarcopenic Obesity OR Skeletal Muscle Loss Obesity） |
| #5 | #1 AND #2 |
| #6 | #5 OR #4 |
| #7 | #3 AND #6 |

**eTable 8. Search Strategy: VIP**

| #1 | M= 肌肉衰减症 or 肌少症 or 少肌症 or 肌肉萎缩 or 肌肉减少症（M= muscle atrophy disorder OR sarcopenia OR hypotonia OR muscle wasting OR muscle reduction syndrome） |
| --- | --- |
| #2 | M= 肥胖 or 超重 （M= Obesity OR Overweight） |
| #3 | M= 糖尿病 （M= Diabetes Mellitus） |
| #4 | M= 少肌性肥胖症 OR 肌少性肥胖症 （M= Sarcopenic Obesity OR Skeletal Muscle Loss Obesity） |
| #5 | #1 AND #2 |
| #6 | #5 OR #4 |
| #7 | #3 AND #6 |

**eTable 9. Agency for Healthcare Research and Quality Checklist for Cross-sectional Studies**

Scoring criteria: 0 = no or unclear; 1 = yes. The total score ranges from 0 to 11. Studies with scores ≥8 were considered low risk of bias, scores of 6–7 were considered moderate risk of bias, and scores ≤5 were considered high risk of bias.

| Item No. | AHRQ checklist item | Score |
| --- | --- | --- |
| 1 | Define the source of information, such as survey or record review. | 0 or 1 |
| 2 | List inclusion and exclusion criteria for study participants or refer to previous publications. | 0 or 1 |
| 3 | Indicate the time period used for identifying patients. | 0 or 1 |
| 4 | Indicate whether or not subjects were consecutive if not population-based. | 0 or 1 |
| 5 | Indicate whether evaluators of subjective components of the study were masked to other aspects of participants’ status. | 0 or 1 |
| 6 | Describe any assessments undertaken for quality assurance purposes, such as test or retest of primary outcome measurements. | 0 or 1 |
| 7 | Explain any patient exclusions from analysis. | 0 or 1 |
| 8 | Describe how confounding was assessed or controlled. | 0 or 1 |
| 9 | Explain how missing data were handled in the analysis, if applicable. | 0 or 1 |
| 10 | Summarize patient response rates and completeness of data collection. | 0 or 1 |
| 11 | Clarify what follow-up, if any, was expected and the percentage of patients for which incomplete data or follow-up was obtained. | 0 or 1 |

**eTable 10. Methodological Quality Assessment of Included Studies Using the AHRQ Checklist**

0: no, 1: yes, overall methodological quality: high (score ≥8), moderate (score 6-7), or low (score ≤5). Items scored: 1) Define the source of information (survey, record review); 2) List inclusion and exclusion criteria for exposed and unexposed subjects (cases and controls) or refer to previous publications; 3) Indicate time period used for identifying patients; 4) Indicate whether or not subjects were consecutive if not population-based; 5) Indicate if evaluators of subjective components of study were masked to other aspects of the status of the participants; 6) Describe any assessments undertaken for quality assurance purposes (e.g., test/retest of primary outcome measurements); 7)Explain any patient exclusions from analysis; 8) Describe how confounding was assessed and/or controlled; 9) If applicable, explain how missing data were handled in the analysis; 10) Summarize patient response rates and completeness of data collection; 11) Clarify what follow-up, if any, was expected and the percentage of patients for which incomplete data or follow-up was obtained

**eTable 11. GRADE Certainty Assessment for the Pooled Prevalence of SO**

| **Outcome** | **No. of studies** | **No. of participants** | **Study design** | **Pooled estimate** | **Risk of bias** | **Inconsistency** | **Indirectness** | **Imprecision** | **Publication bias** | **Certainty of evidence** |
| --- | --- | --- | --- | --- | --- | --- | --- | --- | --- | --- |
| Prevalence of SO among adults with diabetes | 34 | 15,696 | Observational studies | 24% (95% CI: 19%–29%) | Seriousᵃ | Very seriousᵇ | Seriousᶜ | Not serious | Not serious | Very low |

CI, confidence interval; SO, sarcopenic obesity; AHRQ, Agency for Healthcare Research and Quality.

**Explanations**

a Downgraded for risk of bias because most included studies were of moderate methodological quality and eight studies were rated as low quality according to the AHRQ checklist.

b Downgraded for inconsistency because between-study heterogeneity was very substantial, with I² = 98.82%, and prevalence estimates varied widely across studies and subgroups.

c Downgraded for indirectness because the included studies varied substantially in study settings, populations, body composition assessment methods, and diagnostic criteria for sarcopenic obesity.

Imprecision was not downgraded because the confidence interval around the pooled prevalence estimate was considered acceptable. Publication bias was not downgraded because visual inspection of the funnel plot showed no obvious asymmetry and Egger’s regression test did not indicate significant small-study effects.

The pooled estimate represents a single-arm prevalence estimate rather than a comparative intervention effect; therefore, the effect estimate was reported as the pooled prevalence from the random-effects meta-analysis.
